# Dislocation after Hemiarthroplasty due to Insufficiency Fracture of the Superior Acetabulum: A Case Report

**DOI:** 10.1155/2009/257136

**Published:** 2009-07-15

**Authors:** Masahiko Nozawa, Takashi Nishiura, Katsuhiko Maezawa, Keiji Matsuda, Hidenori Morio

**Affiliations:** ^1^Department of Orthopaedic Surgery, Juntendo University Nerima Hospital, 3-1-10 Takanodai, Nerima-ku, Tokyo 117-8521, Japan; ^2^Department of Orthopaedic Surgery, Asakusa Hospital, 1-10-12 Higashi Asakusa, Taitou-ku, Tokyo 111-0025, Japan; ^3^Department of Orthopaedic Surgery, Juntendo University Hospital, 2-1-1 Hongo, Bunkyo-ku, Tokyo 113-8421, Japan

## Abstract

Insufficiency fracture of the superior part of the acetabulum after hemiarthroplasty has not been reported before. Here we report a case of dislocation after hemiarthroplaty due to insufficiency fracture of the acetabulum. In our patient, insufficiency fracture of the ilium immediately above the acetabulum produced obvious evidence of compression and collapse that led to dislocation of the outer head of the femoral component. This patient had *subsequently* been treated with a cementless socket inserted into the fractured acetabulum. We should remember the possibility of insufficiency fracture of the superior acetabulum after hemiarthroplasty, particularly in elderly patients suffering from chronic postoperative pain and disability.

## 1. Introduction

Hemiarthroplasty is a common surgical procedure in elderly patients as a primary treatment for displaced femoral neck fracture or as a secondary procedure after failed internal fixation. We are not aware of any published reports about dislocation after hemiarthroplasty for pseudoarthrosis of a femoral neck fracture due to insufficiency fracture of the superior acetabulum. Here we report a case of this rare type of fracture.

Our patient and his family were informed that data concerning the case would be submitted for publication.

## 2. Case Report

**Figure 1:**
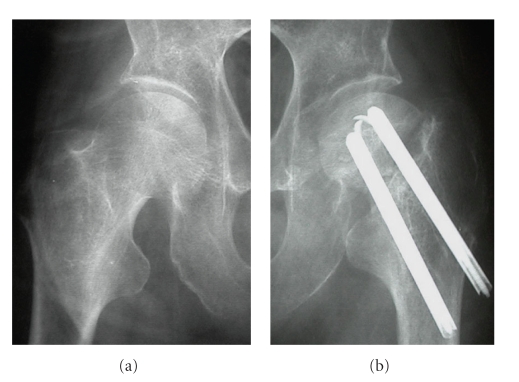
Radiographs show irregularity of the surface of the left acetabulum and osteoporotic changes of the superior part of the left acetabulum in comparison with the right hip. There is an obvious pseudoarthrosis of the femoral neck after internal fixation.

**Figure 2:**
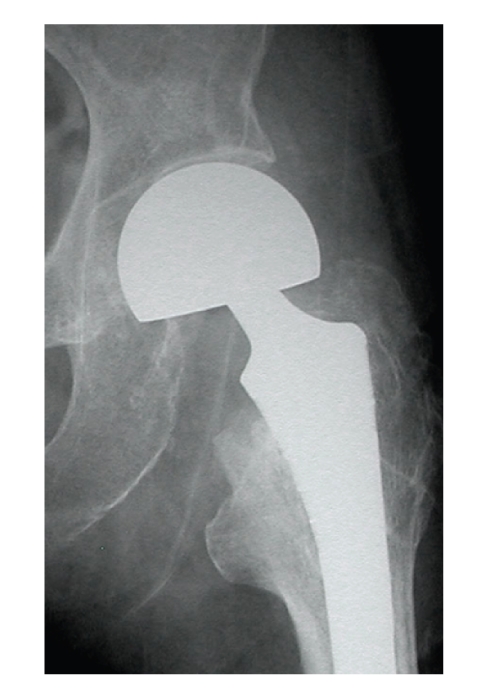
The outer head is set adequately in the acetabulum.

**Figure 3:**
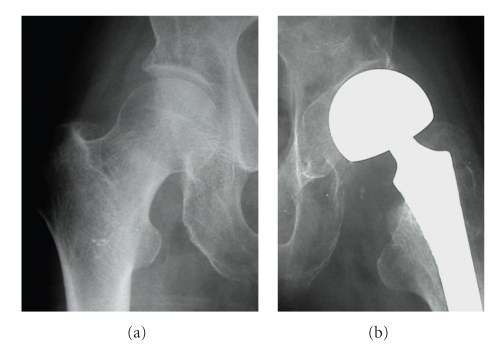
Subluxation of the outer head is apparent. The superior part of the acetabulum shows compression and the inclination of acetabular subchondral bone is increased compared with that on the right side.

**Figure 4:**
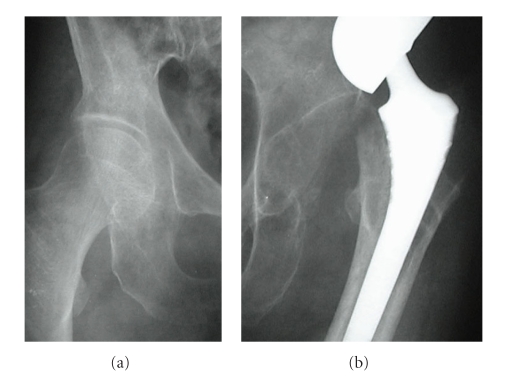
The outer head is dislocated completely. The outer part of the subchondral bone is inclined in the superior direction.

**Figure 5:**
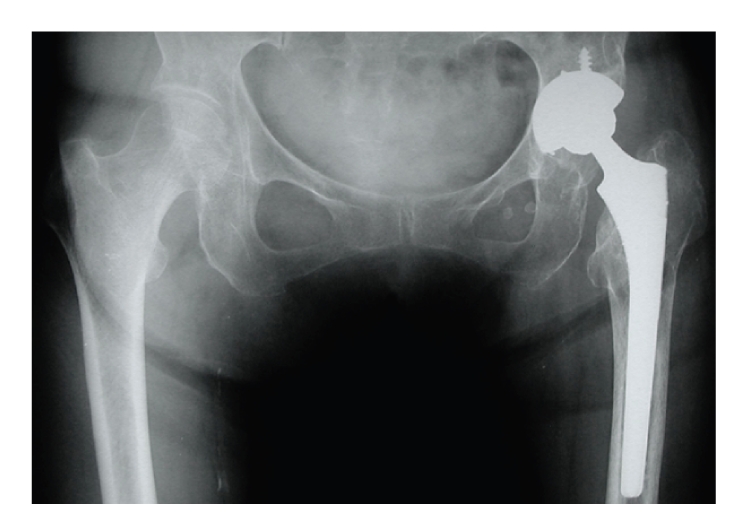
There is no loosening of the acetabular component at three years after revision surgery.

A 74-year-old woman presented to our hospital with pseudoarthrosis of the femoral neck at one year after internal fixation of a femoral neck fracture had been performed with Hansson pins at another hospital. She complained of severe left hip pain and could not walk without two crutches at the first visit. Radiographs obtained at that time showed pseudoarthrosis of a left femoral neck fracture (Figure 1). Laboratory tests revealed no abnormal findings apart from slight anemia. She underwent left hemiarthroplasty with a dual-bearing Natural Hip System (Zimmer Inc.) in November 2004. There was partial abrasion of the cartilage on the acetabular surface at the time of surgery. Stability was satisfactory after reduction of the outer head. We did not recognize supra-acetabular instability during surgery (Figure 2). Walking with full weight bearing was permitted at one week after the operation according to our routine schedule. She had no cognitive dysfunction or dementia. At one month after surgery, she suddenly complained of left hip pain on walking as well as at rest. An antero-posterior radiograph obtained at that time showed slight subluxation of the outer head of the prosthesis and a compression fracture of the superior part of the acetabulum (Figure 3). Collapse at the fracture site progressed over time and the outer head dislocated completely at 6 weeks after surgery (Figure 4). We tried manual reduction under spinal anesthesia, but the procedure failed. Therefore, we removed the outer head and set a cementless socket in the acetabulum. *At the time operation we could find severe erosion of the joint cartilage, however there was no finding of infection*. There was no sign of infection and the stability of this new acetabular component was very good (Figure 5). Full weight bearing while walking was permitted at two weeks after revision surgery. At three years after revision surgery, she has no pain in her left hip joint and walks smoothly with one cane.

## 3. Discussion

The factors contributing to insufficiency fracture include postmenopausal osteoporosis, steroid therapy, radiation therapy, and rheumatoid arthritis. The present patient had required two crutches to walk from one month before her first visit to our hospital. Radiographs obtained at the first examination showed severe supra-acetabular osteoporosis. Therefore, the bone may have had too little elastic resistance to withstand the stress of full weight bearing at one week after revision hemiarthroplasty. Moreover, this patient has suffered from chronic pain and disability before revision hemiarthroplasty, probably resulting in muscle atrophy. Insufficiency fractures are frequently identified in the pelvis and lower extremities of elderly women. Cooper et al. reported that supra-acetabular fractures appear on routine radiographs as hazy sclerotic bands located immediately above and parallel to the acetabular roof [1]. In our patient, insufficiency fracture of the ilium immediately superior to the acetabulum led to obvious radiographic evidence of compression and collapse, with consequent dislocation of the outer head of the femoral component. Periprosthetic acetabular fractures have rarely been reported after total hip arthroplasty [2], and their management has not been standardized. Petersen and Lewallen reported on the management of periprosthetic fracture of the acetabulum after total hip arthroplasty [3]. Kanaji et al. stated that osteolytic insufficiency fractures affecting the medial wall of the acetabulum and associated with loosening of the acetabular cup should be treated by revision arthroplasty [4]. *Additionally, Sánchez-Sotelo et al. reported that severe pelvic osteolysis after THA, if untreated, can lead to eventual periprosthetic pelvic fracture* [5].

*As for the treatments of diplaced femoral neck fractures in the elderly,* *has* *been controversial. Macaulay et al. reported that the decision to perform internal fixation, unipolar hemiarthroplasty, bipolar hemiarthroplasty, or THA must be based on the patient mental status, living arrangement, level of independence and activity, as well as bone and joint quality* [6].

When bipolar hip hemiarthroplasty was introduced, one of its proposed advantages was that the double joint within the implant would reduce the risk of dislocation. The factors predisposing to dislocation after hemiarthroplasty include the use of a posterior approach to the hip, the length of the residual femoral neck, the type of the implant, acetabular dysplasia, muscle imbalance, and neurological conditions such as Parkinsonism, stroke, and dementia. In particular, a posterolateral approach is associated with a significantly increased risk of prosthetic dislocation in patients who have fractures of the femoral neck [7]. *Dislocation of a hemiarthroplasty is associated with a high mortality rate* [8]. Generally, dislocation is treated by closed or open reduction followed by a period of bed rest. If the dislocation is recurrent, surgical treatment might be selected [9, 10]. However, it is recognized that revision total hip arthroplasty is not often an option for these patients in view of its risk. In this present case, we had to perform total hip arthroplasty because other supplementary procedures to stabilize unstable part would not have stabilized the hemiarthroplasty successfully. As a result, we could securely set a new cementless socket into the fractured acetabulum. Reaming of the fractured acetabulum was achieved smoothly without problems, presumably because the compression fracture at the superior part of the acetabulum was comparatively stable. In conclusion, we have to pay attention to the possibility of insufficiency fracture of the superior acetabulum after hemiarthroplasty, particularly in elderly patients suffering from chronic postoperative pain and disability.
